# Effect of thyroid shielding during mammography: measurements on phantom and patient as well as estimation with Monte Carlo simulation

**DOI:** 10.1186/s41747-018-0042-9

**Published:** 2018-06-28

**Authors:** Miriam Pyka, Patrik Eschle, Christian Sommer, Mathias S. Weyland, Rahel Kubik, Stephan Scheidegger

**Affiliations:** 1 Department of Medical Services, Institute of Radiology, Kantonsspital Baden, Baden, Switzerland; 2 Zurich University of Applied Science, ZHAW School of Engineering, Winterthur, Switzerland

**Keywords:** Mammography, Thyroid protection, Radiation-induced cancer, Backscatter, Monte Carlo simulation

## Abstract

**Background:**

During mammography, the thyroid is exposed to scattered radiation from breast tissue and the device. This may increase the risk of radiation induced thyroid cancer.

**Methods:**

We investigated the scatter radiation exposition of the thyroid and the effect of a tailored thyroid protection in phantom and patient as well as by using Monte Carlo simulation (MCS). The protective effect of a modified thyroid protection, the relevance of the protective effect and acceptance by patients have been investigated.

**Results:**

Phantom and patient measurements provided higher values for the surface dose at thyroid position than expected from MCS (phantom 0.32 mGy; patients 0.38 mGy; MCS 0.16 mGy). Phantom measurements indicated scatter contributions from both breast tissue and collimator/tube system. The value found in our patient study is within the range of the literature (0.22–0.39 mGy). The thyroid protection significantly reduced the surface dose but the dose (0.016 mGy) was higher than that expected from the lead equivalent value. However, the impact of the collar to the effective dose was small (< 4%). The collar was not visible on mammograms.

**Conclusions:**

Scatter from the collimator/tube system contributed with 50% to the thyroid dose. Due to the relative small fraction of dose deposited in the thyroid when compared to the mean glandular dose to the breast, a collar is not mandatory in general. Not being associated with the risk of obscuring parts of mammograms, such a collar may be used for young women considering their higher radio sensitivity.

## Key points

- Thyroid dose during mammography was higher than that expected by MCS and the literature
- Radiation from the collimator/tube system contributed approximately 50% to the thyroid dose
- Tailored thyroid protection significantly reduced radiation exposure to the thyroid and was not visible on mammograms
- Thyroid protection is not mandatory but may be taken into consideration for young women

## Background

Mammography is the most important breast examination technique for screening and diagnostic purposes. The abdominal dose during mammography is extremely low [1], making the use of a lead apron for abdominal protection questionable. The thyroid is more exposed to scattered radiation coming from breast tissue (backscatter) and from the device (scatter from the collimator system and leakage radiation).

Radiation exposure of the thyroid, especially at a young age, is a recognised risk factor for the development of thyroid cancer [2, 3]. It was postulated that the increased incidence of thyroid cancer in females might be partially attributed to exposure from medical radiation, including computed tomography and mammography [1, 3, 4]. Of note, due to the low tube voltage used for mammography, absorption in the tissue and backscatter are high. The first effect (caused by the prevalent photoelectric effect) protects the thyroid by the absorption by the overlaying tissue. The second effect (Thomson scattering, high probability of backscatter) contributes significantly to the scatter. Backscatter is dependent on patient anatomy, especially breast size, breast density and, related to this, breast compression.

The dose to the breast was investigated by Hendrik et al. [5, 6] while the thyroid dose and surface dose at thyroid position acquired during mammography were investigated by Sechopoulos [1], Whelan et al. [7], Chetlen et al. [8] and Kunosic et al. [9]. In the study by Whelan et al. [7], the radiation dose to the skin overlying the thyroid for 91 women undergoing routine screening mammography was measured while the study by Chetlen et al. [8] included 207 women. Baptista et al. [10] compared exposition of organs caused by digital mammography and digital breast tomosynthesis by measurements and Monte Carlo simulation (MCS). For a bilateral digital mammography in craniocaudal (CC) view, a thyroid dose of 0.273 mGy was found by these authors.

Ramalho et al. [11] investigated the dose reduction to the thyroid obtained by adopting a standard shielding collar as those commonly used by interventional radiologists. In that case, the problem is that the lower part of the thyroid collar can shadow parts of the breast. Therefore, we tested in this study a modified thyroid protection in form of a collar. Such a collar covers the neck, as all usual interventional collars do, but finishes at the jugulum height. The measurements by Ramalho et al. using a General Electric Senograph unit and an inflatable body phantom showed a reduction of the entrance surface dose from 0.16 mGy without protection to 0.018 mGy with protection. Whelan et al. [7] found a large variability of skin dose values (0.39 mGy ± 0.22 mGy, mean ± standard deviation), resulting in a thyroid organ dose of approximately 0.04 mGy; Chetlen et al. [8] reported doses from 0.05 mGy to 0.82 mGy, with an average value of 0.25 mGy ± 0.116 mGy (mean ± standard deviation). As a consequence, we can assume that the patient anatomical variability has an impact on the scatter dose contribution to the thyroid.

The aim of our study was to measure the dose contribution from scatter to the thyroid and to evaluate the effect of a tailored thyroid protection, using a state-of-the art digital mammography equipment. The following points were investigated: (1) the protective effect of a modified thyroid protection (is there a significant reduction of the surface dose at thyroid position, in particular for a modern digital device with tungsten, instead of molybdenum anode?); (2) relevance of such a protective effect (reduction of thyroid dose compared to the breast dose and effective dose estimated by MCS and exposition parameters of the patient study); (3) acceptance by patients and handling of protective device; and (4) influence of patient-individual parameters such as compression thickness to the thyroid exposure.

## Methods

For the evaluation of the organ doses to the breast, thyroid and ovaries, phantom and patient measurements have been compared with MCS. All measurements have been carried out on a Mammomat Inspiration unit (Siemens Medical Solutions, Erlangen, Germany) using a tungsten anode with rhodium filter. The device was equipped with functions for optimisation of compression (OPCOMP®, Siemens Medical Solutions, Erlangen, Germany) and exposure (OPDOSE®, Siemens Medical Solutions, Erlangen, Germany, Mammomat Inspiration Instruction Manual XPW7-330.620.01.01.01). OPDOSE selects an optimal combination of tube voltage (kV) and anode-filter combination, based on the compression force and compression thickness found by OPCOMP and an automatic exposure control image (short exposition before main image acquisition). With increasing thickness, OPDOSE regulates kV up to reduce time and dose.

### Phantom study

The aims of phantom measurements were: (1) to determine the dependence of scatter on compression-thickness, useful to compare surface dose with MCS and patient study; and (2) to evaluate the spatial/angular scatter contributions.

**Fig. 1:**
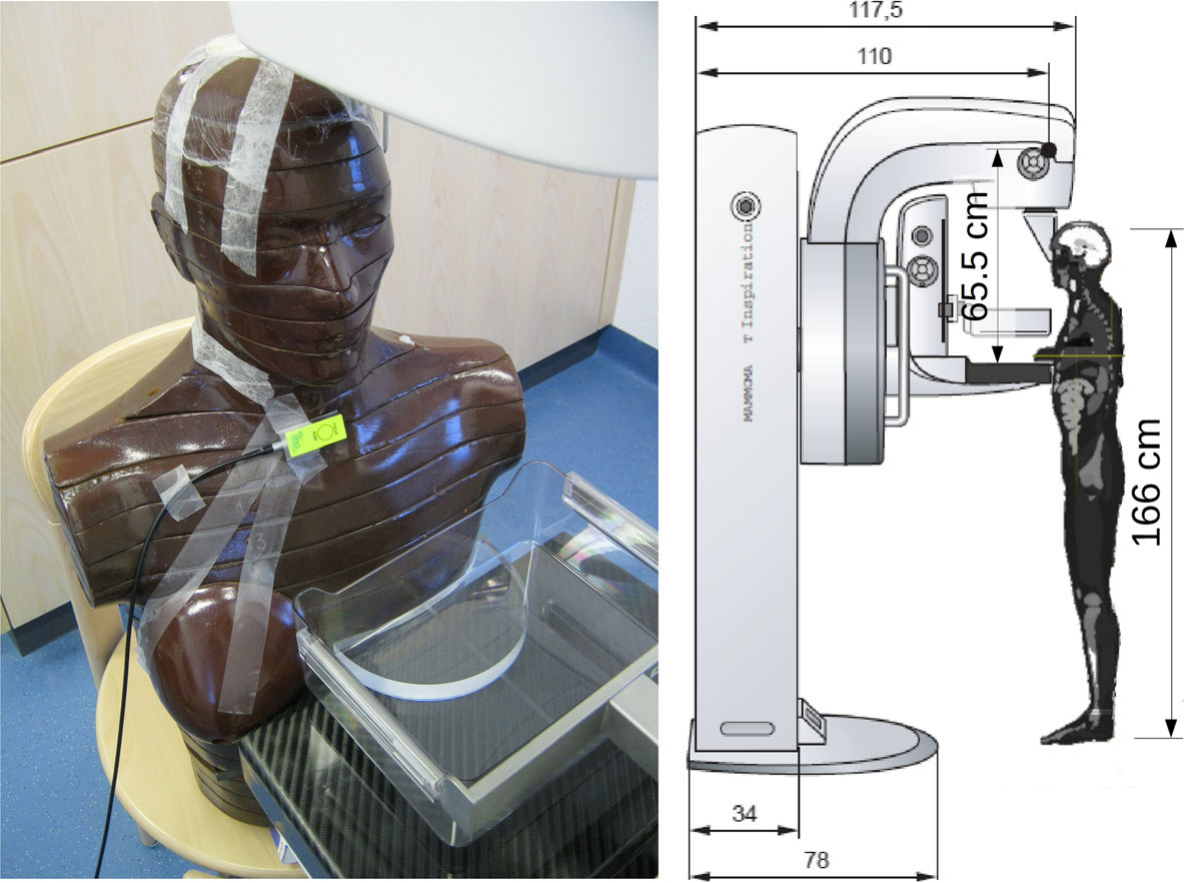
Measurements: on the *left*, with Alderson and breast phantom; on the *right*, geometry for measurements and MCS. The geometry is not completely equal to the situation with real patients since the head of the Alderson phantom cannot be rotated to the side and for patient and phantom measurement, the height of the image unit has to be adapted. The transparent shield, which is between the patient head and the primary beam, was removed to allow a close position of the Alderson phantom, but was used for patient measurements and MCS.

For the first point, an anthropomorphic Alderson phantom [12] (Fig. 1) with a special breast extension for compressed breast has been used (Fig. 1a): slabs of polymethylmethacrylate (PMMA) of 17 × 14 cm in size and thickness of 4, 5, 6 or 7 cm, with the shape of a compressed breast and with breast extension simulating the uncompressed breast. Standard CC and mediolateral oblique (MLO) views were acquired. The dose (air kerma) at the position of the thyroid gland (scatter dose meaning dose related to scattered radiation from breast tissue, collimator, other components and leakage radiation from the unit) has been investigated as a function of the compression thickness by phantom measurements (Alderson phantom with PMMA-slabs for mimicking compressed breasts). The Alderson phantom is designed for radiation therapy and therefore the densities are not exactly tissue equivalent for low x-ray energies [12]. Dedicated MCS of back scatter revealed a correction factor between 1.002 for muscle and 1.03 (28 kVp) or 1.04 (35 kVp) for lead for a scatter angle of 50° (maximum effect at this angle, s. section dosimeter calibration). When the phantom is only used to mimicking the back scatter of a body representing the patient anatomy, this effect can be neglected. The measurements were taken with an RQM solid state detector (IBA Dosimetry GmbH, Schwarzenbruck, Germany) directed to the beam according to Fig. 1 (the RQM sensor does not see the full dose but can indicate the relative increase).

For the second point, to evaluate scatter contributions from breast tissue and collimator system, measurements without Alderson phantom but with PMMA slabs and dosimeters (IBA RQM and re-calibrated Automess SEQ-6R [13], energy range from 18 keV to 3 MeV; section dosimeter calibration) attached to a PMMA plate at thyroid positions have been carried out. To access information about the angular distribution of scattered radiation, measurements have been taken with and without a Pb-shield which covers 180° of the sensitive chamber volume and is directly attached to the dosimeter.

### Patient study

The patient study was intended to investigate the influence of variability of patient anatomy on scattered dose in front of and behind the thyroid protection. It was approved by the Ethical Committee at the Kantonsspital Baden, Switzerland, and written informed consent was obtained from all included patients. Patients scheduled for screening or diagnostic mammography were eligible in the absence of the following exclusion criteria: prior operations of one or both breasts; visible asymmetries; palpable lump; and breast implants. Three patients refused participation. One was excluded after inspection (visible breast asymmetry).

Measurements were taken with a modified thyroid collar having a lead equivalent value of 0.25 mm (Wiroma, Niederscherli, Switzerland). A total of 82 patients were categorised in three breast-size categories based on the CC mammogram of the right breast: 27 large (L-group); 22 medium (M-group); and 33 small (S-group). These criteria were based on a volumetric calculation using compression thickness, anteroposterior and right-left dimensions previously measured in 40 mammograms (unpublished data). Eleven patients in the L-group and one patient in the M-group were studied with the with 24 × 30 cm^2^ paddle; the remaining patients were studied with the 18 × 24 cm^2^ paddle.

**Fig. 2:**
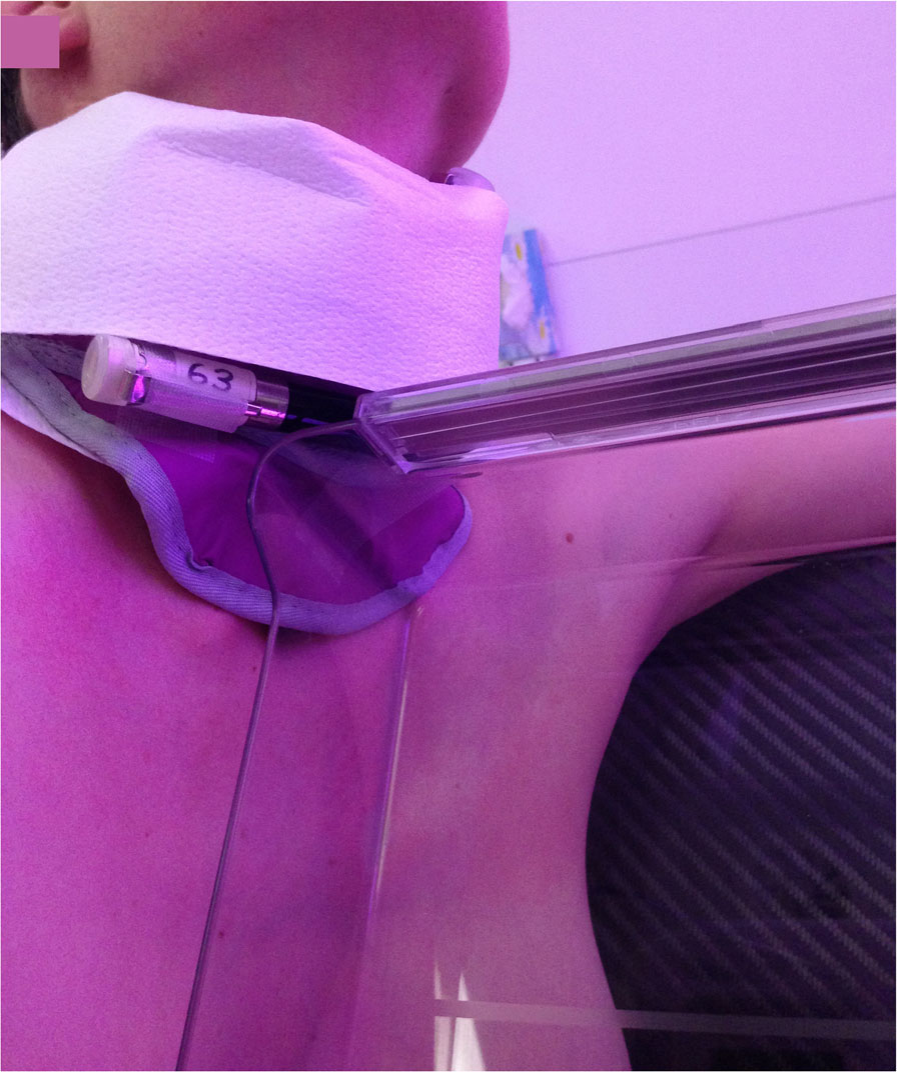
Patient measurement (MLO view) with modified thyroid protection collar and external dosimeter.

The thyroid collar and two SEQ-6R- dosimeters (see phantom study) were fixed, one in front of and one behind the thyroid collar in the neck midline (Fig. 2). For each patient, the measurements were taken during a two-view standard mammography of the left breast. The application of thyroid collar and dosimeters as well as reading of the results after each measurement were always performed by the same examiner. The images were examined regarding quality and artefacts applying the PGMI-criteria (perfect, good, moderate, inadequate) [14], as required for all certified breast imaging centres in Switzerland. The MGD values were calculated automatically for each exposition by the Mammomat software and were registered for each examination.

To evaluate the protective effect (air kerma behind the collar compared to the air kerma value in front of the collar), a paired t-test was used on the full sample as well as on each of the three breast-size categories (L-group, M-group and S-group). For comparisons among groups, a one-way analysis of variance was used, followed by Tukey’s range test. The statistical tests were applied to the dose values measured in front of the collar as well as to those measured behind the collar. Results with *p* values < 0.05 were interpreted as statistically significant. Since we expected large breasts to yield more backscatter, we investigated the relationship between body mass index (BMI) (associated with large breasts) and the air kerma in front of the thyroid protection device.

### Dosimeter calibration

Because of the high sensitivity at specific low-energy radiation and radial isotropy (ability to measure backscatter), we used Automess SEQ-6R dosimeters (energy range from 18 keV to 3 MeV; dose range 0.01–2.00 mGy). All dosimeters were re-calibrated by separated calibration measurement for the radiation quality in use (30 kVp W-Rh target) to the air kerma with a RQM detector (IBA Dosimetry GmbH, Schwarzenbruck, Germany), with Dosimax plus unit, range 500 nGy – 9999 mGy). Energy dependence and linearity were checked by free air measurements with 20 kVp W-target. We compared the back scatter from muscle tissue according to ICRP110 [15] and muscle tissue under a layer of 0.25 mm Pb (collar lead equivalent) and under a layer of 2.5 mm PMMA, irradiated with 28 and 35 keV W/Rh x-rays by MCS. Based on MCS, the effect of the thyroid collar to the backscatter was estimated to be < 3% and therefore, no separated backscatter correction for the collar was applied for patient measurements. It is assumed that the air kerma (in the investigated energy range, this corresponds to the absorbed dose in air and is in the following taken as measure for the entrance surface dose) at the surface in front of the thyroid was representative for the effect on the thyroid dose.

### Monte Carlo simulation

We used the Geant4 simulation toolkit [16]. A numerical voxel phantom was created with the XCAT program [17] (voxel size 2 × 2 × 2 mm^3^). Each voxel was assigned to an organ. Considering the thyroid gland is as an extended organ with an inhomogeneous dose distribution, the dose was calculated based on the real anatomic situation implemented in the voxel phantom. Accumulated dose was calculated by absorbed energy divided by the voxel mass. The x-ray beam was modelled by electrons with kinetic energies of 28 or 35 keV hitting a W-target inclined at 20°. The radiation was filtered with 50-μm Rh according to the Siemens Mammomat Inspiration manual specifications. The beam opening angle was chosen to fully cover the compression plate. In the model, the x-ray head was simplified as a lead cube of 1-mm wall thickness (shielding of 1E-9 at 35 keV) with a rectangular hole in the bottom. Distances and dimensions were modelled according to the Siemens Mammomat Inspiration manual specifications and on-site measurements. The breast tissue was assumed to be a mixture of fat and glandular tissue (ratio 3:2). According to Verdù et al. [18], different breast tissue compositions have been investigated and the uncertainty of tissue composition onto the glandular dose was estimated to be ± 10% for adipose tissue ratios of 40%, 50% and 60%. From the simulated thyroid dose of approximately 100 pGy, we expected a fluence of 59 cm^−2^ through the thyroid. Assuming an area of 4 cm^2^ for the thyroid, we expected 200 photons with an additional uncertainty of 7%. A standard low-energy package was used as recommended in literature [19]. The MCS toolkit was used to calculate all organ doses defined by the ICRP 103 recommendations [20]. The effective dose was calculated on the basis of these organ doses by applying the ICRP 103 model.

## Results

**Fig. 3:**
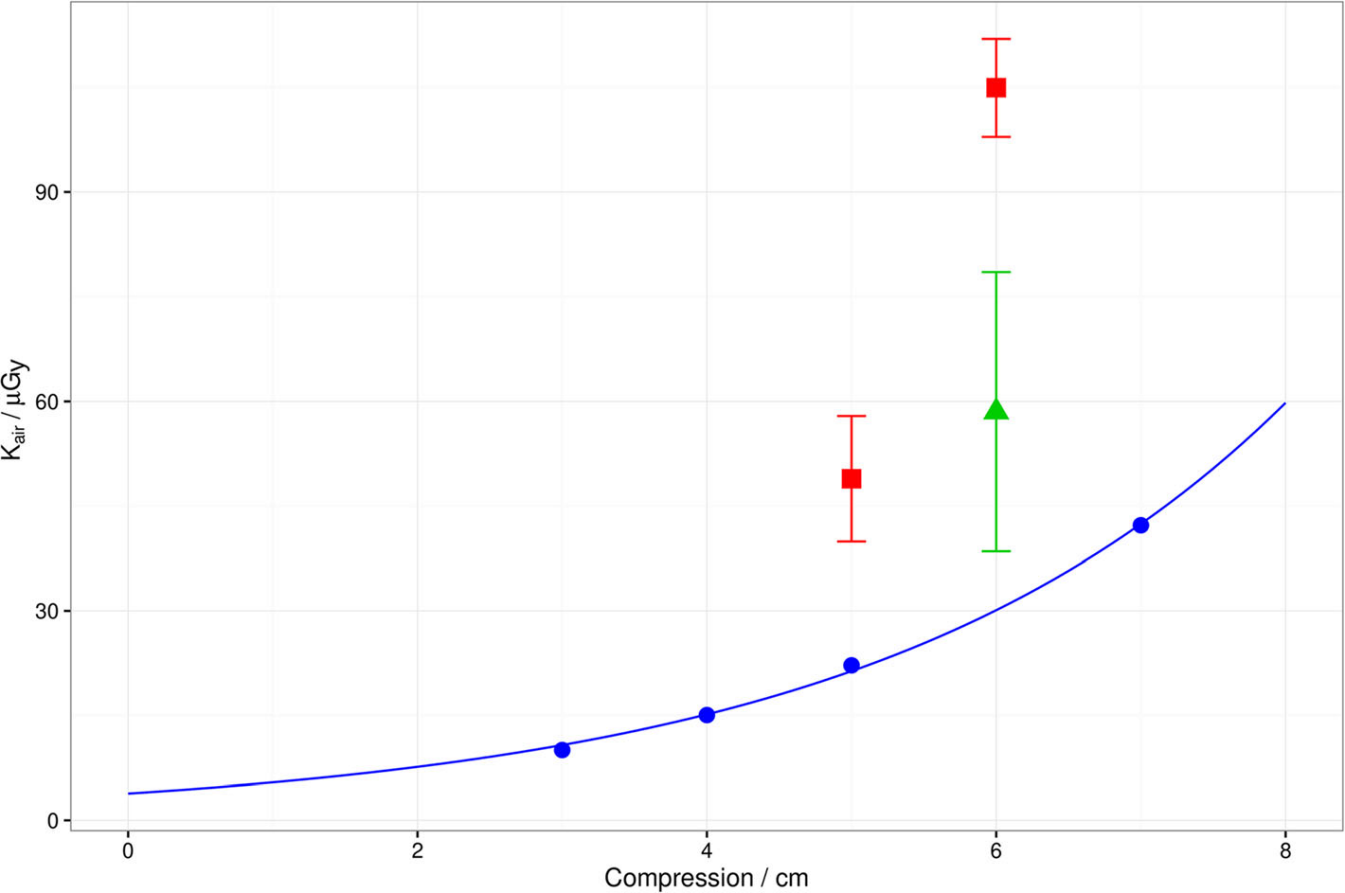
Compression thickness dependence of surface dose (*K*_*air*_ = air kerma) at the position of the thyroid gland (phantom measurements) for one CC view. *Blue dots* represent measures obtained with a PMMA phantom mimicking the compressed breast tissue and the patient’s body represented by an Alderson phantom; the measurements were taken with a RQM sensor directed horizontally to the beam, perpendicular to the beam axis (see Fig. 1). *Red squares* indicate measurements with the same PMMA slab phantom, but without Alderson phantom. The measurements were performed with a SEQ-6R dosimeter. *Green triangle* represents a measurement with SEQ-6R dosimeter using a Pb-shield directed upward to omit scatter and leakage radiation coming from the collimator system. *Error bars* represent standard deviations. The *blue solid line* is an exponential fit to the blue dots: *K*_*air*_ = 3.837*μGy* ⋅ exp(0.3433*cm*^−1^ ⋅ *Compression*).

**Fig. 4:**
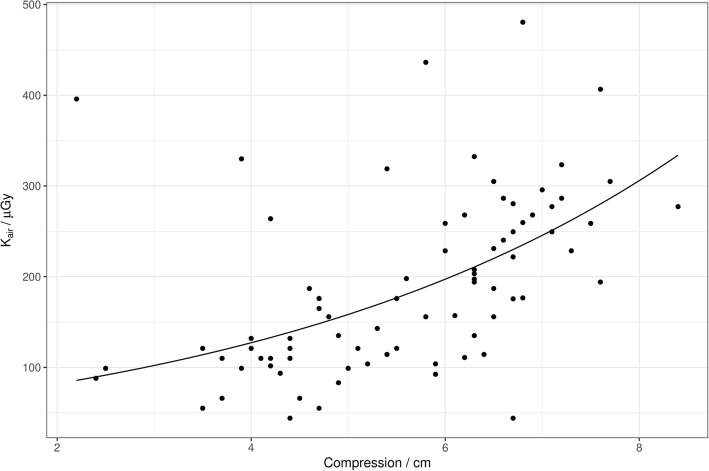
Compression thickness dependence (CC direction) of surface dose (*K*_*air*_ = air kerma = outside dose) at the position of the thyroid gland in patients. The values for air kerma are given for one CC view and one ML view. Tube voltage and tube current × time have been regulated by the automatic exposure control in the range of 26–32 kV and 36–237 mAs, respectively. The *blue solid line* is an exponential fit to the blue dots: *K*_*air*_ = 52.87*μGy* ⋅ exp(0.2194*cm*^−1^ ⋅ *Compression*).

Compared to the patient study, we found a more pronounced increase for the scatter dose with increasing thickness of PMMA slabs (for 7 cm compared to 3 cm factor 4.2 in Fig. 3, blue line). For the air kerma in the patient measurement, we found an average increase clearly below a factor of 4 (Fig. 4; factor 2.4 with exponential fit).

The measurements dedicated to evaluate spatial/angular scatter contributions without Alderson phantom (sensors attached on a thin PMMA plate at thyroid positions) and with a SEQ-6R dosimeter (which has a more isotropic response) revealed higher dose values (red squares in Fig. 3), indicating dose contributions coming from different angles. The green triangle in Fig. 3 represents a measurement with SEQ-6R dosimeter with a Pb-shield directed upward for omitting the scattered radiation and leakage radiation coming from the collimator system. Additional measurements using this Pb-shielding downward (directed to the scatter coming from the PMMA slabs) indicated that both directions contribute more or less equally with a dose of 58 μGy for one CC exposure with 30 kV and 100 mAs. Comparing the exponential fits in Fig. 3 (values for one CC view, measured with RQM-sensor) and Fig. 4 (one CC view and one MLO view, measured with SEQ dosimeter), the air kerma values were clearly higher for the patients. At 6 cm, one CC view results in a dose of 30 μGy (Fig. 3). In Fig. 4, the corresponding value for a single view can be estimated by the half of the value and was 100 μGy. The discrepancy was smaller for the measurement with the SEQ dosimeter in Fig. 3 (for the directed measurement / green triangle, 58 mGy which is slightly more than the half of the patient value).

In Table 1, the exposure parameters for phantom and patient measurements are summarised, where tube voltage and current were a result of the automatic dose control (OPDOSE). The average compression thickness was 55.5 mm for the CC view and 57.1 mm for the MLO view.

**Table 1:** Tube voltage (kV) and tube current – time products (mAs) for phantom and patient measurements

Group / Type	Tube voltage (kV; single value, range, or mean ± standard deviation)		Tube current (mAs; single value, range, or mean ± standard deviation)		Compression thickness (mm; single value, range, or mean ± standard deviation)	
	CC	MLO	CC	MLO	CC	MLO
Breast phantom without Alderson, two different compression thicknesses	28		80		50	
	30		100		60	
Breast phantom with Alderson	26–30	27–30	52–231	51–240	30–70	30–70
Patient L-group	30.2 ± 0.8	30.6 ± 0.9	105.4 ± 26.5	119.6 ± 36.4	67.3 ± 7.8	70.2 ± 9.0
Patient M-group	29.5 ± 0.8	29.7 ± 0.9	101.8 ± 30.9	111.1 ± 41.1	59.4 ± 7.7	60.6 ± 9.5
Patient S-group	28.0 ± 0.9	28.2 ± 0.9	79.3 ± 28.3	82.7 ± 20.1	43.9 ± 9.3	44.9 ± 7.4
All patients	29.1 ± 1.3	29.4 ± 1.4	93.6 ± 30.6	102.0 ± 35.9	55.5 ± 17.7	57.1 ± 13.9

**Fig. 5:**
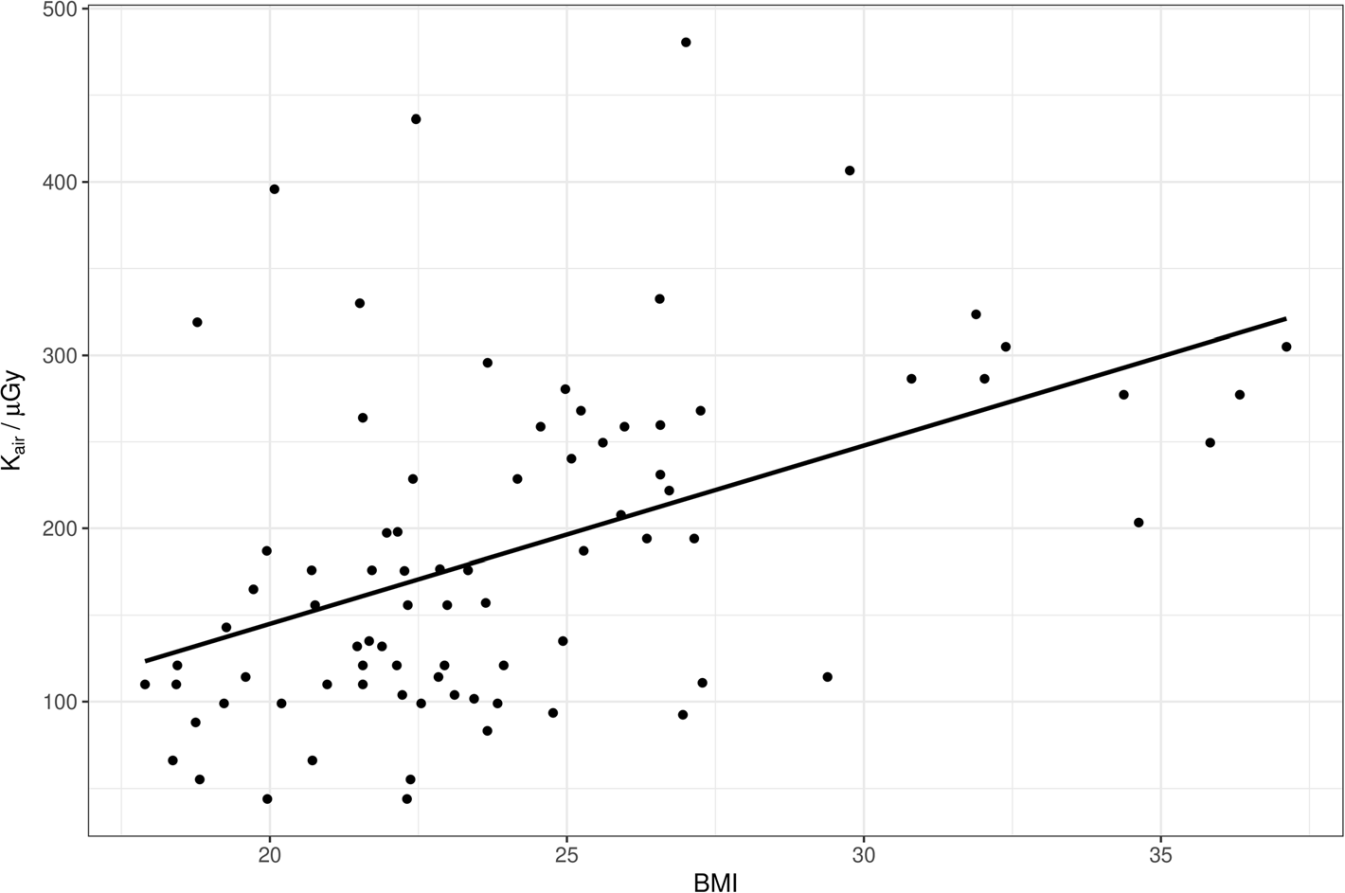
*Scatter plot* of air kerma (*K*_*air*_) in front of the thyroid protection device with body mass index and so, approximately, with breast volume. The BMI correlates less (*R* = 0.48) to backscatter than compression thickness because even large breasts may sometimes have only a thin compression thickness. Therefore, the determining factor for backscatter is compression thickness: *K*_*air*_ =  − 60.75*μ*Gy + 10.29*μ*Gy ⋅ *BMI*.

The scatter plot showing the relation between the air kerma and body mass index (BMI) is presented in Fig. 5. It reveals a weak correlation (Pearson *r* = 0.48).

In Table 2, the mean air kerma values (scattered dose values for patients) in front of and behind the collar are displayed. The difference in measured dose was significant (*p* < 0.001), but behind the collar, a higher dose value than that expected from the lead equivalent value was observed (6–10% of the dose values in front of the collar, at 35 kV; < 0.1% was expected for 0.25 mm Pb and direct beam transmission). No significant difference in the dose values measured behind the collar among L-group, M-group and S-group was found (*p* ≥ 0.332).

**Table 2:** Left: mean dose (air kerma ± standard error) in front of (outside dose) and behind thyroid protection (inside dose), groups L = large, M = medium, S = small; measured values for 1× CC and 1× MLO left breast. Right: Results of ANOVA and Tukey’s range test. Measured values for one craniocaudal view and one mediolateral oblique view of the left breast. Left: paired t-test. Right: ANOVA and Tukey’s range test

Group	Outside dose (mGy)	Inside dose (mGy)	*p* value	Comparison	Outside dose *p* value	Inside dose *p* value
All (*n* = 82)	0.187 ± 0.011	0.016 ± 0.002	< 0.001	Overall	< 0.001	0.364
L-group (*n* = 27)	0.249 ± 0.016	0.020 ± 0.004	< 0.001	L-group vs M-group	0.019	0.813
M-group (*n* = 22)	0.182 ± 0.019	0.017 ± 0.004	< 0.001	L-group vs S-group	< 0.001	0.332
S-group (*n* = 33)	0.141 ± 0.014	0.013 ± 0.003	< 0.001	M-group vs S-group	0.190	0.762

The dose values presented in Table 2 cannot be compared directly to the thyroid doses, since the values in this Table are air kerma values. For organ doses, the absorption of the tissue under consideration of the incident beam angle has to be applied and the dose values have to be converted from air to tissue. The MCS calculation with a beam quality for a tube voltage in the range of 28–35 kV and an average breast entrance dose (automatic calculation for each exposition by the Mammomat software and available in DICOM data for each examination) of 3.6 mGy observed in the patient study (3.4 ± 1.4 mGy for the CC view and 3.7 ± 1.8 mGy for the MLO view) resulted in a thyroid dose from 4.3 μGy (28 kV) to 5.8 μGy (35 kV) for a two-view bilateral mammography. The corresponding air kerma values on the surface at thyroid position calculated by the MCS were 158 μGy (28 kV) and 162 μGy (35 kV) for a two-view bilateral mammography with the same average entrance or MGD as found in the patient study. This was approximately the half of the average air kerma outside value in the patient measurement shown in Table 2 (187 μGy for left breast, 374 μGy for a full four-view mammography). The air kerma value measured with PMMA breast phantom was approximately 80 μGy for one CC exposition and 5.55 cm compression (estimated by interpolating the data / red squares shown in Fig. 3). Thus, a two-view bilateral mammography therefore sums up to approximately 320 μGy, a value close to the surface dose found in the patient study. Table 3 presents an overview of surface dose values originating from different studies.

**Table 3:** Summary of surface and thyroid dose values found in the different studies

	Monte Carlo simulation	Phantom measurement	Patient study
Surface dose at thyroid position, 4 views, current study, Siemens Mammomat	158 μGy (28 kV) 162 μGy (35 kV)	~ 320 μGy (compression 5.55 cm)	374 μGy (29 kVp, 98 mAs per view average, 0.95 μGy/mAs)
Surface dose at thyroid position 4 views, Chetlen et al. [8], Senograph Essential, GE Health Care			240 μGy (right lobe) 250 μGy (left lobe) (29 kVp, 58 mAs per view in average, 1.06 at 29 kVp)
Surface dose at thyroid position 4 views, Whelan et al. [7], Senograph DMR, GE Health Care			390 μGy (average 1.05 μGy /mAs)
Surface dose (4 films), Kunosic et al. [9]			220 μGy
Surface dose at thyroid position, 4 views. Estimated by the ratio of our MCS and Sechopoulos et al. [1]	38–126 μGy		
Thyroid dose resulting from bilateral digital mammography examination in CC view; Baptista et al. [10]	273 μGy		
Surface dose at thyroid position, Ramalho et al. [11], GE Senograph		160 μGy (compression 4.5 cm)	

For a two-view bilateral mammography, the MCS resulted in an effective dose of 214 μSv (28 kV) and 234 μSv (35 kV) for the average entrance dose found in the patient study. The contribution of MGD to the effective dose is 98.6% (28 kV) and 98.0% (35 kV). The relative dose contributions to the different organs are given in Table 4. The highest exposition (of a patient of the L-group) would lead to a maximum effective dose of 300 μSv.

**Table 4:** Relative dose contributions to the effective dose, calculated by Monte Carlo simulation

Organ group / Tissue	Relative contribution to effective dose (no leakage radiation from x-ray head)	
	35 kV	28 kV
Red bone marrow	0.40%	0.30%
Stomach wall	0.04%	0.02%
Bladder	0.00%	0.00%
Liver	0.01%	0.00%
Cortical bone	0.03%	0.02%
Oesophagus	0.01%	0.00%
Salivary gland	0.09%	0.09%
Skin	0.54%	0.50%
Brain	0.00%	0.00%
Lung	0.69%	0.36%
Breast	97.99%	98.55%
Thyroid	0.10%	0.08%
Gonades (female)	0.00%	0.00%
Intestine	0.01%	0.01%
ICRP103_Rest	0.08%	0.06%

The application of the thyroid collar was quick and that did not slow down the workflow. It took about 3–5 s and was easily done by an experienced radiology technician. The patients in our study did not complain of compression or pain, but most of them thought that to have a thyroid collar around the neck in an already emotional situation was rather uncomfortable.

The image quality of mammograms was found to be state-of-the-art without differences regarding the right mammograms without thyroid collar and the left mammograms with thyroid collar. In none of the cases the collar was visible on the images. In three cases, one retake was necessary because the dosimeter was visible in the image (these measures were not included in the dose calculation).

## Discussion

The thyroid protection used in this study significantly reduced the air kerma, which was assumed to be proportional to the scattering dose to the thyroid. Due to scatter, the air kerma behind the protective collar was higher than expected from the lead equivalent value (16 ± 2 μGy in average for all patients; see Table 2). According the thickness of the lead of 0.25 mm, the incident beam was expected to be attenuated by a factor of 15.10^–5^. The dose we estimated behind the collar is in agreement with the findings reported by Ramalho et al. (18 μGy) [11]. Measurements with phantoms revealed scatter contributions (50%) from upside (collimator system) and downside (backscatter form breast and detector system, 50% of the total contribution; see Fig. 3, green triangle), resulting in air kerma values up to > 100 μGy for 6-cm compression at the surface of thyroid position. This value is clearly greater than the values found in the MCS but corresponds to the values we found in the patient study. In the MCS, 85% of the dose came from backscatter of breast and detector system and only 15% was caused by collimator scatter.

In comparison to the patient study, only the half value of the air kerma at the surface was found by the MCS. As indicated by the red squares and green triangle in Fig. 3, approximately half of the measured air kerma was contributed by scatter or leakage radiation coming from the tube/collimator system. To cover the collimator scatter more appropriately, the collimator system should be implemented in more detail in the MCS model. In front of the thyroid, for a two-view bilateral mammography, the experiment measured an air kerma of 374 μGy while the MCS measured only 162 μGy. The experimental value was higher than the MCS by a factor of 2.3. We estimate the influence on the effective dose by increasing all 15 organ doses according to ICRP 103 [20] except on the directly irradiated breast by this factor 2.3. The effective dose increases by < 4%. This is due to the large fraction of total effective dose in the irradiated breast (~ 97%).

The thyroid dose calculated by MCS with the parameters according to the average patient in the patient study was in the range of 4–6 μGy (depending on the tube voltage) for a two-view bilateral mammography and 1–1.5 μGy for one CC exposition. Sechopoulos et al. reported a thyroid dose for each mammographic view of 0.016–0.045% of the MGD, depending on the view needed and exposure parameters/beam quality [1, 3]. In our patient study, with an average MGD of 0.9 mGy for one CC view, this would result in a thyroid dose of 0.144–0.405 μGy. Of note, the MGD found by our patient study is in accordance with data reported by Bosmans et al. [21]. For a two-view bilateral mammography, this sums up to a thyroid dose in the range of 0.7–1.6 μGy. Based on this value and the ratio between thyroid dose and air kerma found by the MCS model, we can expect an air kerma of 38–126 μGy. In Table 3, this range is compared to surface dose values found by other studies. Whelan et al. reported a surface dose (skin overlying the thyroid) of 0.39 ± 0.22 mGy [7]. This surface dose value is similar to the results of our patient study and is higher than the value found by Chetlen et al. (0.250 ± 0.116 mGy) or by Kunosic et al. (0.220 ± 0.01 mGy). In our patient study, the average tube voltages are comparable to those reported in the patient study by Chetlen et al. [8]. The average compression thickness was slightly below the values found by Chetlen et al. [8] (58–59 mm for the CC view and 62–63 mm for the MLO view). The trend of the BMI in Fig. 5. is in line with Chetlen et al. [8] and can be attributed to the fact that large breasts can sometimes still be compressed to low thicknesses. Our results for the surface dose in the patient study (374 ± 11 μGy in average) correspond to a thyroid dose of approximately 10 ± 2 μGy when applying a conversion factor derived by the comparison of the surface dose values of MCS (4.3 μGy) and measurement, both at 28 kV (air kerma[patient] / air kerma[MCS] = 374 mGy / 158 mGy = 2.367). This is in agreement with the phantom study reported by Ali et al. (9.5 μGy measured by thermoluminescence dosimeter in an ATOM phantom) [22]. Baptista et al. [10], using MCS, found a thyroid dose of 0.273 mGy for the CC view with a corresponding breast MGD of 2 mGy. Compared to our MCS and those reported in the literature, this result is surprisingly high for the organ dose value.

In contrast to the change in MGD found in patients by Bosmans et al. [21], we found a more pronounced increase for the scatter dose with increasing thickness of PMMA-slabs (for a 7-cm compared to a 3-cm compression thickness). In our phantom measurements, the higher density of the PMMA plate and, related to this, the higher mAs and kV values selected by the automatic dose control led to a more pronounced rise of the dose with increasing compression thickness. We should note that the PMMA slabs do not perfectly simulate the breast glandular tissue. Therefore, patient measurements are important. The patient measurements do support the phantom study and take into account the real anatomic variability in women (morphology and mobility of the neck, difference in breast density, and difference in thyroid–breast distance). However, the variability found in our study is smaller than that reported by Whelan et al. [7].

The effective dose we calculated by MCS for the parameters according to the average patient in the patient study was approximately 0.2–0.3 mSv, lower than the effective dose reported by Ali et al. (0.326 mSv) [22], reflecting the tendency for differences between MCS and measurements. The contribution of the thyroid was small compared to the breast dose. From this point of view, the relevance of the use of a protective collar may be questionable in a routine clinical setting.

It is important to have data about real patient settings to advise and reassure patients in case of questions about the necessity and usefulness of a thyroid collar. The use of a thyroid protection that does not obscure part of mammogram (as we proposed) leads to a similar reduction in the thyroid dose compared to a standard thyroid protection device. However, with the sensitivity of the thyroid gland for ionising radiation being more relevant in childhood and young adulthood [2, 3], the proposed thyroid protection may be taken into consideration especially for young women, outside the regular screening setting.

## Abbreviations

BMI: Body mass index
CC: Craniocaudal
MCS: Monte Carlo simulation
MLO: Mediolateral oblique
PMMA: Polymethylmethacrylate
